# Interactions and non-magnetic fractional quantization in one-dimension

**DOI:** 10.1063/5.0061921

**Published:** 2021-09-15

**Authors:** S. Kumar, M. Pepper

**Affiliations:** Department of Electronic and Electrical Engineering, UCL, Torrington Place, London WC1E 7JE, United Kingdom and London Centre for Nanotechnology, 17-19 Gordon Street, London WC1H 0AH, United Kingdom

## Abstract

In this Perspective article, we present recent developments on interaction effects on the carrier transport properties of one-dimensional (1D) semiconductor quantum wires fabricated using the GaAs/AlGaAs system, particularly the emergence of the long predicted fractional quantization of conductance in the absence of a magnetic field. Over three decades ago, it was shown that transport through a 1D system leads to integer quantized conductance given by N·2e^2^/h, where N is the number of allowed energy levels (N = 1, 2, 3, …). Recent experiments have shown that a weaker confinement potential and low carrier concentration provide a testbed for electrons strongly interacting. The consequence leads to a reconfiguration of the electron distribution into a zigzag assembly which, unexpectedly, was found to exhibit quantization of conductance predominantly at 1/6, 2/5, 1/4, and 1/2 in units of e^2^/h. These fractional states may appear similar to the fractional states seen in the Fractional Quantum Hall Effect; however, the system does not possess a filling factor and they differ in the nature of their physical causes. The states may have promise for the emergent topological quantum computing schemes as they are controllable by gate voltages with a distinct identity.

The ground-breaking investigations performed within the last 60 years or so exploring the fundamental quantum aspects of low dimensional electron transport occurred as a result of technological developments. By an interesting scientific coincidence, the Field Effect, where applying an electric field between two conductors separated by an insulator to vary the carrier concentration, was first investigated by C. F. Mott, the father of the immensely well-known solid state theorist N. F. Mott. This was at the suggestion of Mott's supervisor J. J. Thomson shortly after his discovery of the electron in 1897. As Mott used metals, no effect was observed, and it was only in the 1960s that the quality of semiconductor–insulator interfaces improved to such an extent that successful experimentation was possible, particularly using the Silicon Metal Oxide Semiconductor Field Effect Transistor (MOSFET).

The use of MOSFET devices was pioneered by the IBM group who showed that the inversion layer of the device was two-dimensional (2D) and found Landau levels resulting from magnetic quantization of the 2D density of states resulting in Shubnikov–de-Haas oscillations.^1^ The device was subsequently used for detailed studies of Anderson Localization, such as Variable Range Hopping^2^ and the nonexistence of true metallic states in 2D.^3^ A particularly noteworthy development was the discovery of the Quantum Hall Effect (QHE) in 1980;^4^ the accuracy and extent of the quantization of the Hall conductance in units of e^2^/h were not anticipated by physics, which did predict the geometrical independence of the Hall conductance when the Fermi energy was between Landau levels.^5,6^ The QHE is most easily understood on the edge state model combined with localization of states in the tails of the Landau levels, which are located away from the edges. The net result is that conduction in the bulk of a 2D system is rendered negligible at low temperatures, the edge states support one-dimensional (1D) conduction as the electrons at each edge cannot be backscattered, and the conductance is quantized taking values of Ne^2^/h, where N is the number of non-communicating edge states at the Fermi level.^7^ The large distance between the states at the two edges results in an infinitesimal probability of electron tunneling between the two and the remarkable accuracy of the quantization.

The development of growth techniques, particularly modulation doped Molecular Beam Epitaxy (MBE), allowed the formation of much lower disorder structures and a correspondingly higher mobility of the 2DEG formed at the interface of GaAs/AlGaAs. The materials advancement led to the discovery of the interaction based Fractional Quantum Hall Effect (FQHE) when the disorder element was substantially reduced.^8^ Use of low disorder GaAs/AlGaAs heterostructures makes possible a detailed study of one-dimensional (1D) electron transport. The simplest method of obtaining a 1D system is by electrostatically confining the electron/hole gas by split or patterned gates, initially used in silicon,^9,10^ but more successfully with the GaAs heterostructure.^11,12^ The flexibility of using split and patterned Schottky gates allows a constriction, termed a quantum point contact or quantum wire, to be formed in which the confinement can be altered by negative gate voltages (positive if holes are confined); as the confinement potential is relaxed, the 1D channel becomes wider, and the quantized levels drop in energy, so changing the conductance by 2e^2^/h as they populate. The quantized conductance is N2e^2^/h, where N is the number of allowed 1D subbands, and the factor 2 is due to spin degeneracy. The quantization occurs in the ballistic regime where the sample length is less than the elastic scattering mean free path, of significance, for later work, the length is also less than the inelastic, phase coherence length. Early work showed the 1D conductance quantization with high mobility electrons in GaAs heterostructures;^13,14^ now this effect has been observed in other materials.^15–18^

1D conductance quantization, measured in the two-terminal mode, is not accurate as in the case of the QHE, as positive and negative momentum states are spatially coincident enhancing any backscattering, and measurements of resistance will include the 2D regions between the quantum wire and the Ohmic contacts. The basic feature of one-dimensionality giving rise to the quantization is that the density of states of a level at the Fermi energy in 1D can be written as N(E) = 1/*h*v, where v is the Fermi velocity. The conductance due to a narrow band of states traveling at the Fermi velocity v is then 1/h, which leads to the conductance quantization; the transition between the spin degenerate and single spin cases is most easily achieved by the application of a parallel magnetic field. A perpendicular magnetic field will further squeeze the energy levels leading to depopulation^11^ and the limiting case of the QHE in a narrow 2DEG.

Figure 1 shows a timeline of the evolution of ballistic quantum phenomena in the transport of 2D/1D electrons. It is shown that the Integer and Fractional QHEs were discovered in 1980 and 1982, respectively,^4,8^ and the 1D density of states and quantum correction in 1986; the quantization of conductance in a 1D quantum wire was demonstrated in 1988. The following year, in 1989, non-linear transport in 1D quantum wire was experimentally observed based on the theoretical prediction by Glazman and Khaetskii,^19^ which initiated the concept of subband spectroscopy in 1D systems. One of the most surprising manifestations of many body physics, the 0.7 conductance anomaly,^20^ was investigated in detail in 1996, and in 2009, experimental demonstration of the 1D Incipient Wigner Lattice was performed;^21^ this effect contained many intriguing associated phenomena^22,23^ that subsequently led to the discovery of non-magnetic fractional quantized conductance in 1D systems, based on holes^24^ (2018) and electrons^25^ (2018–2019). It was almost 36 years between this discovery and that of the FQHE principally due to advancement in fabrication technology, availability of high-quality material, and refinement in the ability to manipulate electron wavefunctions at the boundary of the 1D–2D transition.

**FIG. 1.**
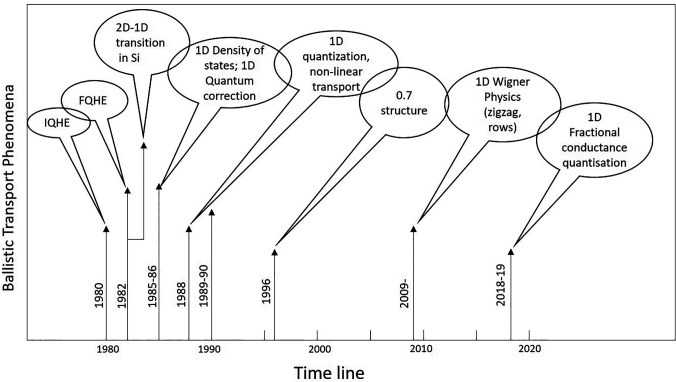
A timeline of the evolution of ballistic transport phenomena in the transport of the 2D/1D systems.

In this article, we provide our perspective of recent developments in the quantum physics of 1D systems and future possibilities in the field. Figure 2(a) shows the evolution of the 1D physics based on the control of the carrier concentration and confinement with the ability to manipulate many body effects within the system. Some of the effects highlighted in this figure will be discussed in this Perspective.

**FIG. 2.**
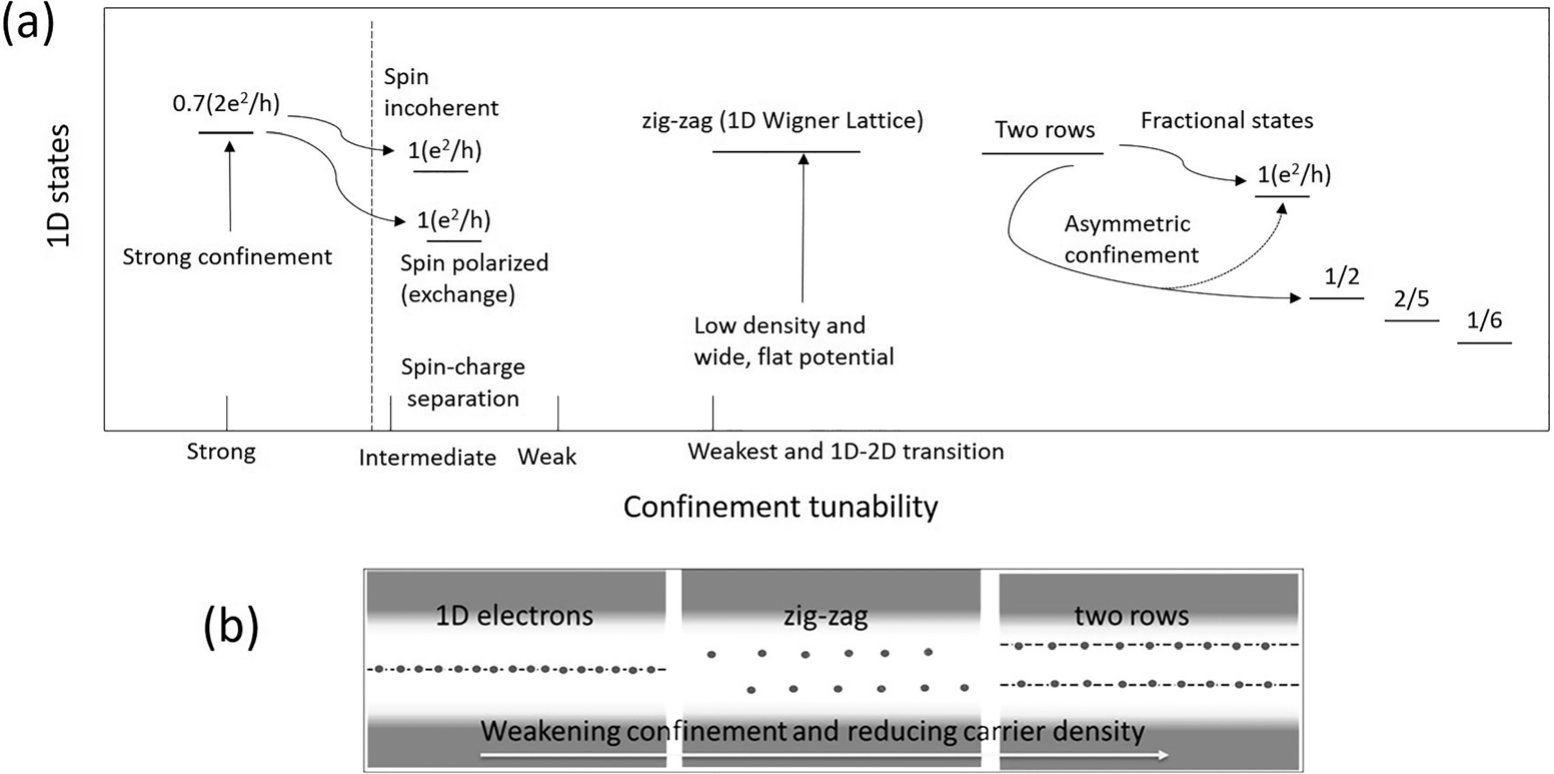
(a) The evolution of 1D physics in the quantum transport of quantum wires as a function of tuning the confinement potential. It is shown here how a variety of 1D states emerge out when the interaction between the 1D electrons and the externally applied potential confining them are varied. (b) The emergence of 1D Wigner lattice as a function of the changing carrier concentration and confinement potential.

The theory of quantization of conductance results in integer values of conductance and is unable to explain a feature that occurs below 2e^2^/h; this can be in the form of conductance structure often a plateau and is termed the 0.7 although it can occur anywhere between 0.6 and 0.8 in units of 2e^2^/h.^20,26^ A striking aspect of the 0.7 is that the application of a parallel magnetic field, B, causes the feature to drop smoothly to e^2^/h, indicative of a complete spin polarization; a similar effect will occur with a perpendicular magnetic field, but as this rapidly leads to the QHE, so a parallel magnetic field configuration is preferred. A spin splitting at zero B is found below both the first and second 1D subbands accompanied by an enhanced g value. Subsequent calculations also pointed to the existence of a zero B lifted spin degeneracy;^27–31^ the direction of which can be slowly varying on the timescale of transport through the 1D region. This can give rise to a spin dependent back-scattering.

The observations that the 0.7 occurs due to the partial spin polarization in the ground state in a 1D channel would appear to contradict the theorem of Lieb and Mattis^32^ that the ground state of an infinite 1D system must be non-magnetic. It is not clear if this theorem holds in the presence of an applied voltage or for a nanostructure that is quasi-1D. Evolution of the conductance plateaus with the dc source-drain voltage and magnetic field indicates that nearby the 0.7, the two spin levels split, with an occupied lowest level and a non-degenerate upper spin level pinned above the source chemical potential. Current in the channel is now due to the transmission of the lower spin level giving 0.5(2*e*^2^/*h*) and partial transmission of the upper spin level; a consequence is an enhanced *g* value that increases with population of the lowest level. Eventually, as the carrier concentration increases, the upper level is pulled below the source chemical potential, and the normal 2*e*^2^/*h* plateau is obtained. As the temperature decreases, so the upper spin level moves down in energy to become close to, and eventually at, the source potential, so becoming degenerate, resulting in the increase and eventual disappearance of the 0.7 into the 2*e*^2^/*h* plateau at the lowest temperature.^33^

The 0.7 decreases toward the fully spin-polarized value of 0.5^34^ as the carrier concentration is reduced resulting in suggestions that there is an absence of the upper spin level. In a similar manner, it has been suggested that as the fully spin polarized value is obtained for longer channels, there is an absence of minority spin carriers that can tunnel;^35^ the result is the formation of an enhanced spin gap.

Thermopower measurements have been taken on the 0.7 structure. The thermo-electric power exhibited by 1D devices has shown that there is a general agreement with the Mott formula ( $\pi^{2}{\frac{k_{B}T}{3e}.\partial G/G\partial \mu }^{}$, where *μ* is the chemical potential, *G* is the conductance, *k_B_* is the Boltzmann constant, and e is the electron charge) for the integer quantized plateau. The thermopower exhibited by 1D devices shows a peak corresponding to each riser in quantized conductance agreeing with the Mott formula.^36^ However, at low carrier concentrations, there are deviations associated with many body effects as a flat 0.7 plateau might be expected to show a zero, or minimal, thermopower, as found and predicted for integer plateaus, due to the lack of dependence of conductance on the carrier concentration. On the contrary, the thermopower is high, indicating a breakdown of the underlying conditions leading to the Mott formula.^36^ It is possible that as spin polarized electrons emerge toward the cold end of the sample, in a thermopower measurement, there will be a transition to the spin degenerate situation and a decrease in the Fermi energy. The heat emission due to this process will raise the temperature of the cold end and produce an increase in measured thermopower. Significantly for understanding the 0.7, a thermal conductance plateau was found at 0.5 when the conductance was in the region of the 0.7 indicating the formation of a spin gap.^37^

Other investigations include compressibility which produced results on the behavior of the 0.7 in agreement with that expected for a spontaneous spin polarization and not in accordance with Kondo predictions.^38^ Measurements showed a reduction in shot noise on both integer and 0.7 plateaus; the Fano factor indicated two different channels of transport; these could be identified as the two unequally populated spin channels expected for a spin polarization.^39^ An interesting aspect of the behavior of higher integer plateaus was found when a crossing of opposite spin levels was induced by a parallel magnetic field and a spontaneous spin polarization was found.^40^ A theory of the process^41^ had success in accounting for the behavior, in particular a breakaway conductance plateau that differed according to whether it was approached from high or low magnetic fields; this hysteresis arose from the exchange between spin populations induced by the magnetic field.

It has been suggested that the 0.7 may be a Kondo peak due to the formation of a localized state in the channel caused by backscattering.^42,43^ It is known that resonance features in the conductance are strong in the presence of back scattering,^44^ and these can be produced by impurities. The discussion on whether the 0.7 arose from a localized state in the channel^43^ or another mechanism was investigated using conventional split gate channels with the ends of the gates slightly curved to reflect carriers and produce a bound state. The results showed that moving the conducting channel to one side enhanced back-scattering and produced an excellent example of a Kondo peak in conductance; however, this was superimposed on the 0.7, showing that the two had different physical origins. In the absence of the imposed back-scattering, a Kondo peak was entirely absent.^45^ A similar conclusion that the 0.7 is fundamental and not related to localized states was obtained by scanning probe measurements.^46^ Scattering in the channel due to impurities or defects can produce considerable mesoscopic structure in the conductance,^47^ which can then give rise to localization and a Kondo structure.^48^ These results indicate the necessity of utilizing clean disorder free channels for the observation of spontaneous spin polarization, which has also been observed in hole systems.^49^ Recent theory has proposed that there is a spin polarization that slowly rotates as carriers progress through the channel; this effect can explain the experimental findings including the observed zero bias anomaly.^30,50,51^ It has been known for some time that in one-dimension, all states are Anderson localized.^52,53^ Essentially, the probability of an electron being forward scattered, and diffusing through a 1D device decreases to zero as the system size increases. However, the devices used in studies described here are of order 1 μm in length, and unless the sample is deliberately disordered, the number of scattering events is limited. This can be of order 1 or 2 as in Smith *et al.*^47^ Consequently, the only effect of localization in the characteristics of the conductance is the Kondo effect described earlier.

There are several other spin effects in addition to the 0.7. One of the characteristics of a Luttinger liquid is that the strong interaction gives rise to a separation of charge and spin transports and spin-charge separation.^54–56^ However, in a low-density system, the mutual repulsion keeps carriers widely separated; the exchange interaction is minimized and is less than the thermal energy; consequently, the spin direction is random and no longer has a defined value.^57^ Such a spin incoherent Luttinger liquid has been identified by a suppression of the 2e^2^/h plateau to e^2^/h as the spin quantum number is no longer operative, and states must be singly occupied.^58^

A characteristic of an unequal spin population is as with the 0.7 a tendency to ferromagnetic behavior; an enhanced and oscillating g value was found as levels were filled in the presence of a magnetic field. This effect is essentially a magnified version of the behavior in the absence of a magnetic field when the spin repulsion is due to the exchange only.^59–62^

When the applied source-drain voltage, V**_sd_**, is small, there is no difference between the conductance (I/V_s_**_d_**), and the differential conductance (dI/dV**_sd_**), the integer plateaus appearing at 2Ne^2^/h, N = 1, 2, 3, …. However, as V**_sd_** increases, so the plateaus in conductance disappear, but with differential conductance, the integer plateau in 2e^2^/h occurs at e^2^/h and then 2e^2^/h, a relationship which can be derived simply.^63–65^ The two plateaus are separated in energy by eV**_sd_/**2, reflecting the lifting of the momentum degeneracy, which allows a direct measurement of the energy in the channel as a function of gate voltage. When the momentum degeneracy is completely lifted and electrons flow in one direction, a plateau at e^2^/h would be expected, but this drops to level out at e^2^/2h, i.e., 0.25(2e^2^/h). Such a result implies that both the spin and momentum degeneracy are removed, and the stream of electrons possesses ferromagnetic ordering although no defined direction. Alternatively, the measurement of the conductance, rather than the differential, is proportional to the band filling that reflects the repulsion between the spin levels. The spin polarization in the non-linear regime has been directly observed and utilized for spin filtering and control in an electron focusing experiment.^66^

Quantum calculations^67,68^ have shown that a single row of electrons will become progressively distorted as the interaction (confinement potential) increases (decreases), to form a zig-zag array, eventually splitting into two separate rows, from right to left as confinement weakens, Fig. 2(b), i.e., a transition from bonding to anti-bonding states. The two lines of electrons are similar to the lines considered for cold atoms and ladder compounds,^69,70^ and rotational chiral currents may result from such a configuration. Matveev, Meyer, and co-workers^71,72^ investigated the different exchange processes in which the spectator electrons are allowed to relax. As the interaction increases, the nature of the ground state varies from a two-particle exchange based on a particle in each row to a four-particle ring; however, the energies of the different rings are close, making difficult a precise determination of the ground state. The transition from one row to a zig-zag is predicted to occur when the separation, in units of the Wigner–Seitz radius, r_s_, is less than 0.78, and the resultant zig-zag is stable until r_s_ is 1.75 when more rows form. Eventually, the zig-zag configuration gives way to a more complex arrangement of many rows to form a Wigner Lattice, a conclusion also reached by classical calculations;^73^ here, the stability has been investigated as a function of confinement potential.^74^ The calculations showed that the only allowed second order transition between one and two rows is for a parabolic confinement potential.

By locating a gate over the channel separated by a dielectric from the split gates, it is possible to provide a flexible control by directly altering the carrier concentration as well as the width of the 1D channel. Experiments^21–23^ at a low 2D carrier concentration, <5 × 10^10^ cm^−2^, and an electron mobility of ∼4 × 10^6^ cm^2^/V s showed that as the confinement weakened, the 2e^2^/h plateau either completely (crossing) or partially disappeared (anticrossing). These two effects are shown in Figs. 3 and 4. The device schematic is shown in the inset of Fig. 3(a). Figure 3(a) shows the effects of weakening confinement where the left-hand corresponds to strong confinement with the carrier concentration (initially 2 × 10^11^ cm^−2^) reduced by the top gate voltage, V_tg_, with weaker confinement, for each successive plot of conductance vs split gate voltage, V_sg_. When the split gate threshold voltage is near −3.1 V, the first plateau at 2e^2^/h has weakened considerably compared to the plateau at 4e^2^/h. Moving further to the right, the first plateau is restored, but as seen, the curves become much closer, which indicates a stronger coupling to the split gates; this is expected if the electron wavefunction were to be more laterally extended across the channel. Interpretation of the data is simplified by the examination of the colorscale plot, Fig. 3(b), obtained from the transconductance (the gradient of the conductance) of the data in Fig. 3(a); here, the black lines correspond to the risers between plateaus, and the red corresponds to the plateaus. In the region of strong confinement, top left, there are clear plateau regions between the black lines; the first narrows as the confinement is progressively weakened—moving toward bottom right. At a point near −3.1 V corresponding to the transition in the conductance, there is an anticrossing of the energy levels and a transition in the ground state. Measurements on another sample, Fig. 4, show that the first plateau has completely disappeared [Fig. 4(a)]; the transition may be an anticrossing smeared by disorder or a crossing; the former ground state subsequently crosses the second excited state as seen in both conductance plot and the colorscale plot, Fig. 4(b), of transconductance of data in Fig. 4(a). Such measurements have been repeated in the presence of a 12 T magnetic field in the plane of the 2D electron gas; the anticrossing is very clear, albeit all the values of conductance are reduced by a factor of 2 due to lifting of spin degeneracy [Figs. 4(c) and 4(d)]. However, there is considerable complexity in a series of crossings and anticrossings, which arises from the added variable of spin doubling the number of levels.^22^ Such results have been observed with many different samples^21–23^ with different preparation conditions and from different semiconductor growth centers. We conclude sample independence although high disorder can obscure observation.

**FIG. 3.**
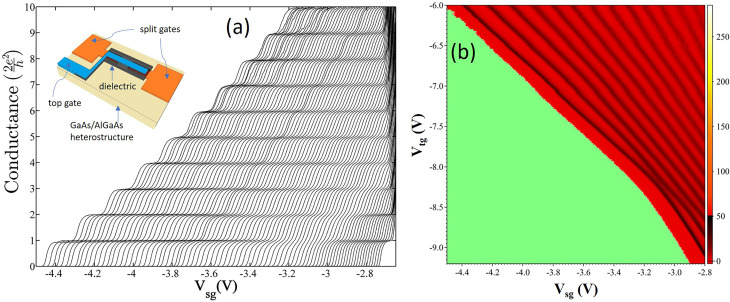
(a) Conductance characteristics of a 1D device as a function of applied top gate voltage [−6.0 (left) to −9.2 V (right)]. Inset shows schematic of a top gated, split gate device defined over a GaAs/AlGaAs heterostructure; here, split gates are shown in orange, the top gate in blue, and the dielectric separating them is shown in gray. (b) A transconductance plot (dG/dV_sg_) of data shown in (a) illustrated as a colorplot, where dark regions represent the conductance risers and the red regions are the conductance plateaus.

**FIG. 4.**
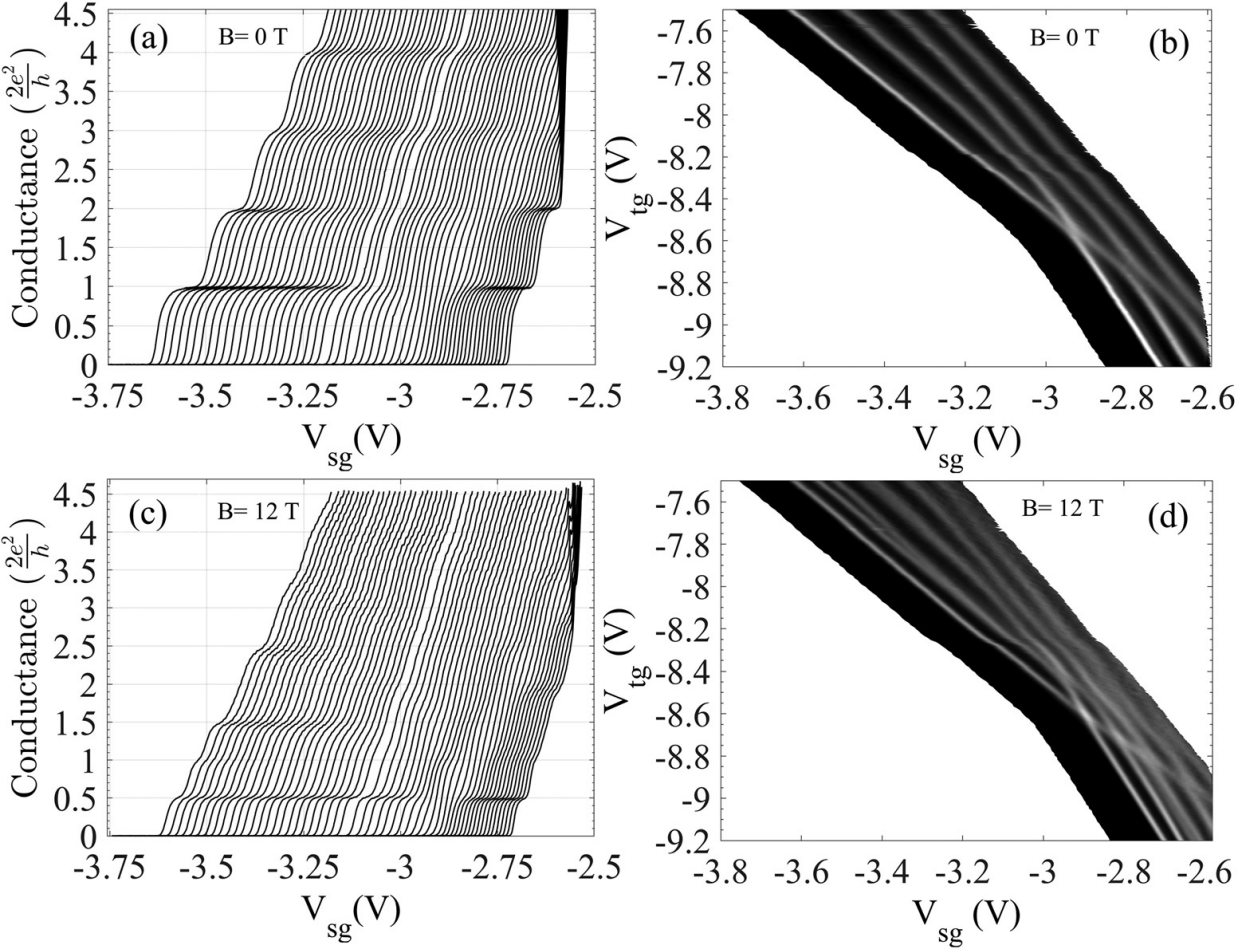
(a) Conductance characteristics of a different 1D device as a function of top gate voltage [∼−7.5 (left) to −9.2 V (right)]. (c) Conductance characteristics in the presence of in-plane magnetic field of 12 T showing Zeeman splitting and crossing of the ground state with the higher subbands as the confinement weakens. (b) and (d) show transconductance plots represented as grayscale plots for the data in (a) and (c), respectively, where gray regions represent the conductance risers, and the dark, black regions are the conductance plateaus. Adapted from Ref. 22.

A further series of measurements, Fig. 5, was conducted on GaAs based 1D quantum wires where the top gate was grounded to simplify the electrostatics. In this measurement, the split gates were asymmetrically biased moving from left to right in the conductance plot.^75^ It was noticed that the effect that was produced by the symmetrical confinement potential in Figs. 3 and 4 was reproduced by applying an asymmetric confinement potential even in the absence of a top gate voltage. As seen, the plateau at 2e^2^/h was significantly weakened on enhancing the asymmetry in applied confinement potential, and at around V_B_ ∼ −3.1 V (V_B_, voltage on one of the split gates, see Fig. 5 caption for further details), so resulting in anticrossing of the ground and first excited states. A different sample where there was no physical top gate present over the split gates was also measured; a colorplot of the transconductance data is shown in Fig. 6. Dual anticrossing (AX) effects were observed, and expected, when the confinement potential was varied from highly asymmetric to symmetric to again highly asymmetric by independently controlling voltages on the split gates.^75^

**FIG. 5.**
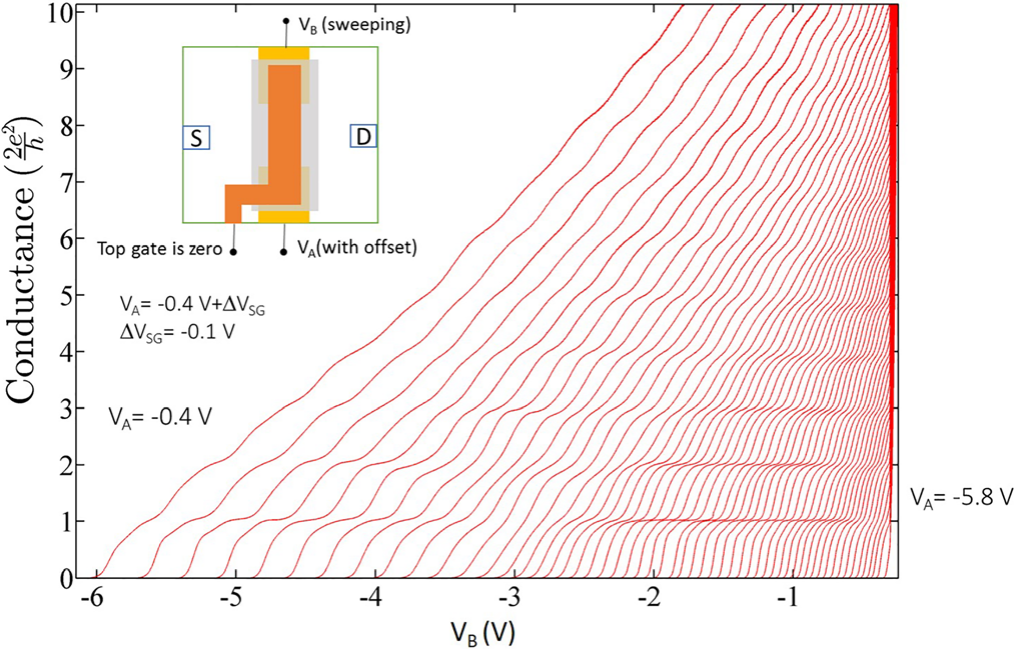
Conductance characteristics measured by sweeping voltage on gate B, V_B_, as a function of increasing negative offset voltage (ΔV_SG_ = −0.1 V) on gate A, so that V_A_ ranges from −0.4 (left) to −5.8 V (right). Here, the confinement potential was made highly asymmetric by holding gate B at a fixed voltage, while gate A was being swept. The inset shows a device schematic where split gates are shown in yellow and a top gate over the split gates in orange. Adapted with permission from Kumar *et al.*, Appl. Phys. Lett. **118**(12), 124002 (2021). Copyright 2021 American Institute of Physics.

**FIG. 6.**
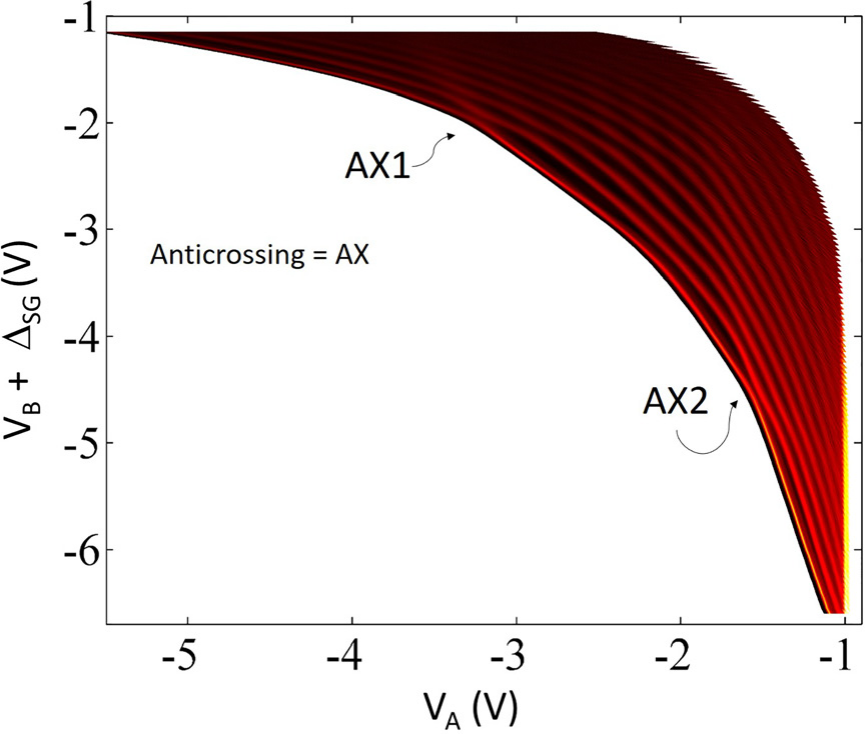
A colorplot of transconductance data [see Fig. 3(a) in Ref. 75 for conductance plot] showing the evolution of two sets of anticrossing of the ground and first excited states, one at V_A_ and V_B_ + ΔV_SG_ (−3.3 and −2.0 V) and the other at V_A_ and V_B_ + ΔV_SG_ (−1.6 and −4.5 V). Adapted with permission from Kumar *et al.*, Appl. Phys. Lett. **118**(12), 124002 (2021). Copyright 2021 American Institute of Physics.

In these measurements, it was shown that in a wide 1D quantum wire, manipulating the confinement potential results in tailored many body effects that produce an Incipient Wigner Lattice when the 1D system is near the transition to 2D. It is termed an Incipient Wigner Lattice as the electron repulsion determines the particular configuration of the electrons as would occur in a 2D Wigner Lattice. Normally, discussion of the behavior of a confined system assumes that there is a lateral quantization of the wavefunctions arising from the spatial confinement. However, if the system width is W, then the ratio of Coulomb energy to confinement energy is ∼W; so at large widths, and especially low carrier concentrations, the energy levels are determined by the Coulomb effects. As the system width increases, confinement weakens; the wavefunction with two centers of charge drops in energy faster than the original ground state where the charge is peaked in the center.^22,75^ This results in the new ground state being formed from the original first excited state although it would be expected that the node would disappear on reducing the energy.

Electron focusing has been used to observe the splitting of the ground state into two corresponding to the formation of the Incipient Wigner Lattice. Focusing comprises injection of electrons into a 2D electron gas subjected to a weak transverse magnetic field, which bends the injected electrons around and focusses them into a narrow detector.^76^ The first resonance occurs when the magnetic field is given by B = 2ħk_F_/eL, where k_F_ is the Fermi wavevector, L is the separation of the injector and detector, and e is the electron charge, so that the separation of injector and detector equals the cyclotron diameter. The assumption is that the electrons are injected from a central point although with broadening; if the electrons are now injected from two points, then the resonance peak is split into two. The situation is shown in Fig. 7, where in (a), an experimental diagram shows the injector and detector, and (b) and (c) illustrate the focusing results (see Fig. 7 and caption for further details). The top plot in Fig. 7(c) shows the resonance focusing signal taken with the injector in strong confinement regime; the consecutive plots on going down correspond to a gradually weakened symmetric confinement potential. The central line of electrons splits into two as the confinement weakens, which results a split in the focusing peak, Fig. 7(b).^77^ The distance between the two injection points is obtained from the known parameters and is approximately 3/4 of the channel width. Such behavior is fully consistent with the establishment of a zig-zag. By applying bias to the detector, it is possible to introduce a spin selectivity to the resonance, which has been used to probe spin texture.^77^

**FIG. 7.**
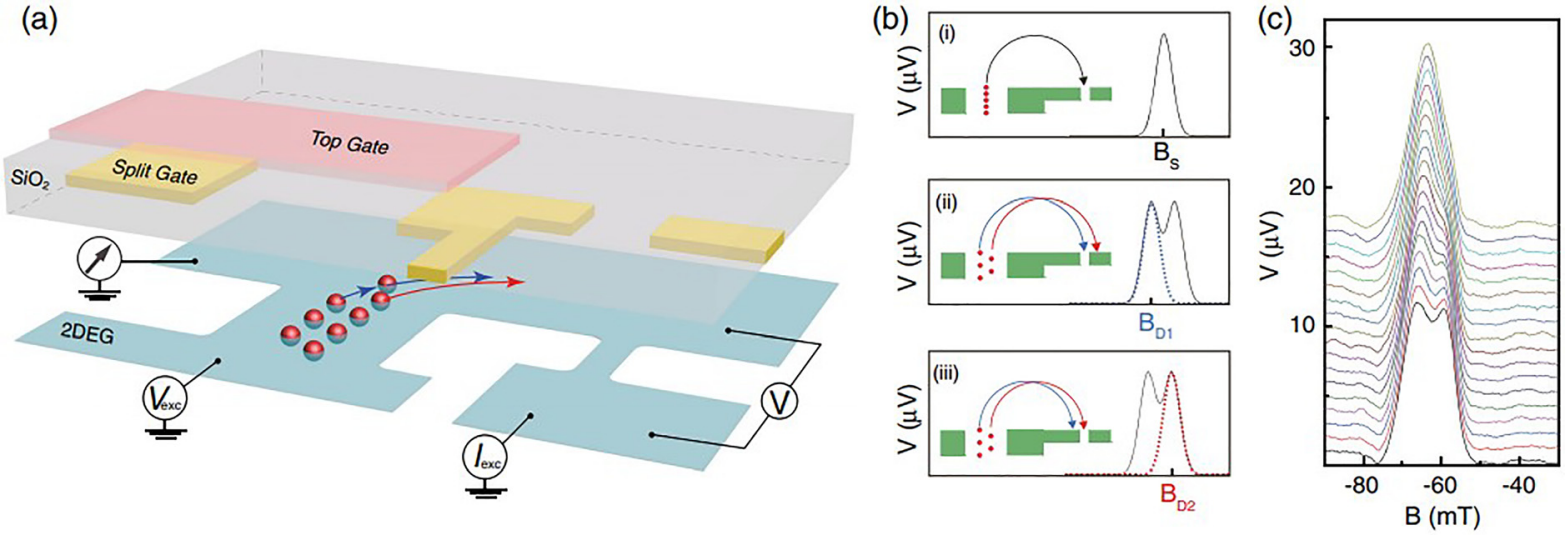
Imaging the zigzag Wigner crystallization formed in a 1D channel using transverse electron focusing. (a) Schematic of a focusing device consisting of a confinement-tunable quantum wire, as injector, on the left, formed by a pair of split gates (shown in yellow) and a top gate (shown in pink) separated by a SiO_2_ insulator. A pair of split gates on the right act as a detector. (b) Illustration of the focusing peaks and their corresponding cyclotron motions. A single row of electrons will produce a single peak as shown in (i) when focused into the detector, whereas electrons in a double row are focused into the detector at two different magnetic fields [(ii) and (iii)], giving rise to a split in the focusing peak. (c) Electron focusing graph when the confinement was tuned from being strong, the top trace, to weak, the bottom trace, which is the regime where the 1D electrons have localized in the 1D Wigner lattice. Adapted with permission from Ho *et al.*, Phys. Rev. Lett. **121**(10), 106801 (2018). Copyright 2018 American Physical Society.

Now we discuss the theory of fractional quantization of conductance in the 1D/2D systems, followed by a mechanism based on the 1D zigzag chain of electrons. The recent experimental results on non-magnetically induced fractional quantization of conductance in the 1D channel will subsequently be discussed. The first theory of non-magnetically induced fractional charges was by Su, Schrieffer, and Heeger (SSH)^78^ (see for a review), was constructed for 1D polymer chains where an alternation in the bonding arrangement could produce a defect with charge e/2. However, as polymer chains are highly disordered, this prediction has not been observed, although in many respects, a ladder compound can be envisaged as two chains that are connected.^69,70^ It has been suggested that the Anomalous Hall effect and Quantum Spin Hall effect in Topological Insulators could produce fractional charges. At present, these have not been observed.^79^

Thouless *et al.*^80^ considered a band structure with a magnetic field applied and showed the existence of the Integer QHE without Landau levels, an example of the Hofstadter Butterfly. Subsequently, Haldane^81^ discussed a half filled honeycomb band structure with zero, net magnetic field arising from a field which reversed every plaquette; the Integer QHE was predicted as a consequence of the breaking of time reversal symmetry. A detailed consideration of Chern insulators has evolved^82^ based on band insulators in which quantization, both integer and fractional, is predicted as a function of the Chern number, which is essentially a path integral around a unit cell. This is essentially the equivalent of the Landau index in a continuum model. Fractional quantization in these materials in the asbsence of magnetic field has not yet been observed.

Calculations and simulations using lattice models have resulted in predictions of odd denominator fractions in the absence of a magnetic field.^83–89^ The tight-binding calculations were based on a high ratio of bandgap to bandwidth, typically 20 to 50; the electron energy was flat with increasing numbers of carriers. Wavefunction overlap only reached as far as second nearest neighbors, and in that respect, the system resembled Landau Levels where the principal energy in the system is the electron–electron repulsion with limited hopping distance. The various models all reached similar conclusions in which a square lattice with flat bands showed minima in energy at 1/3 and 1/5 filling factors.^86^ Similar behavior has been found with hard core bosons^89^ and, in principle, higher temperatures than the liquid Helium range are possible.

Ring exchange was considered in relation to the FQHE shortly after its discovery,^90^ and it was suggested that such a process in the 2D electron gas could explain the fractional quantization. This suggestion created discussion as in the absence of a quantizing magnetic field, ring exchange could increase the electron energy, rather than produce a minimum,^91^ and it was not pursued further in that context. It was suggested that the dominant exchange was due to the large number of electrons participating, and that this could lead to a fractional charge. Prior to this, ring exchange was known to play an important role in the properties of quantum solids such as helium 3, where it was first shown that an odd number of particles in the exchange process leads to a spin polarized configuration. The system was regarded as comprising cavities, atoms, separated by ducts through which exchange tunneling occurred.^92^ In a similar way, an optical field produces cyclic currents and edge states in a cold atom ensemble.^93^ It is possible that a cyclical ring current is a way of lowering the ground state energy by reducing the effects of the confinement, which determines the ground state; the correlated motion produces a cyclic current around the ring, resulting in a lowering of the energy and a gapped state at particular fractional conductance values. There is a certain resemblance here to the vortex state in the FQHE. Figure 8 shows this situation, where the prevailing rotation is clockwise, although it could be opposite, and the two triangles could be in anti-phase. This suggestion is clearly different from the continuum in which the FQHE is found or the lattice theories as the existence of a zigzag configuration can allow a cyclical motion of the carriers. The implications of such a model are that the electrons form their own lattice due to both decreased confinement and the electron–electron interaction. Tunneling through the ducts could create a cyclic current and decreased confinement energy leading to a gap at the Fermi energy.

**FIG. 8.**
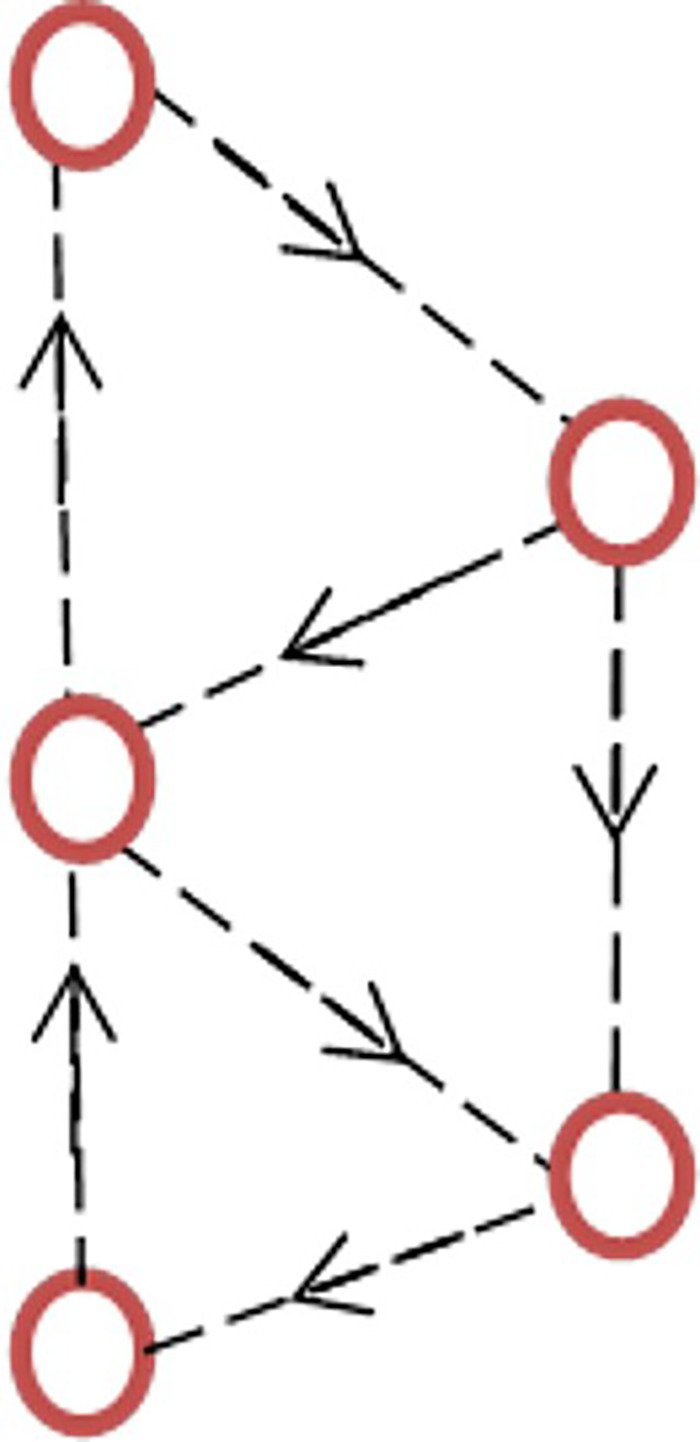
An illustration of cyclic current in a zigzag 1D electron configuration leading to observations of non-magnetic fractional quantized conductance.

1D hole conduction in high mobility germanium grown on germanium–silicon^24^ showed that at very low values of the carrier concentration (2D density ∼ 10^10^ cm^−2^), the first and second integer plateaus disappear and are replaced by fractional plateau at/near (1/2)e^2^/h and (1/16)e^2^/h. In the presence of a high parallel magnetic field, the plateau at (1/2)e^2^/h dropped to (1/4)e^2^/h, indicating that it was spin degenerate at zero magnetic field, and the plateau at (1/16)e^2^/h strengthened, implying that it was spin polarized at zero field.

A search for such non-magnetic fractionally quantized conductance was performed with high mobility GaAs based heterostructures^25,94^ with low values of electron carrier concentration and a tuned asymmetry in the confinement potential. This was successful for 2D values of the carrier concentration near ∼10^10^ cm^−2^, which is much lower than that normally used for investigations into 1D behavior. For weak, symmetric confinement a plateau at (1/2)e^2^/h was present; however, if the confinement potential were asymmetric and weak, then additional fractional plateaus emerged, of which the most prominent and stable were 2/5, 1/2, and 1/6. In Fig. 9(a), the main figure shows 1/2 obtained in an asymmetric confinement potential, and the inset shows 1/2 obtained in a symmetric confinement potential, the only fraction in this configuration. The 2/5 and 1/6 that are shown in Fig. 9(b) were obtained with an asymmetric confinement potential. A 2/3 plateau was frequently found with a slight slope, and a plateau was often present near 3/5 but slightly below the exact fractional value. The flatness of the most stable plateaus from the various samples measured was found to be 1 in 10^4^ over the plateau length for the flattest parts of the 1/6, 1/2, and 2/5 states, as shown in Fig. 10. The length and absolute error from the accurate value of plateaus for these fractions is over 2 (error 0.8%), 3.3 (error 0.2%), and 3.4 mV (error 0.2%). In Fig. 10, sweeps from left to right is for an enhancement in the asymmetry in the applied confinement potential. The 1/6 fractional state is well estabalished and the curves cluster around the 1/6 until the asymmtry in the confinement potential is further enhanced and resonant type structure similar to theory in Fig. 2(a) in Ref. 95 is found. One consistent feature of the fractional results is the disappearance of the 1 and 2 integer plateaus, indicating that the interaction is determining the electron states when confinement is weak.

**FIG. 9.**
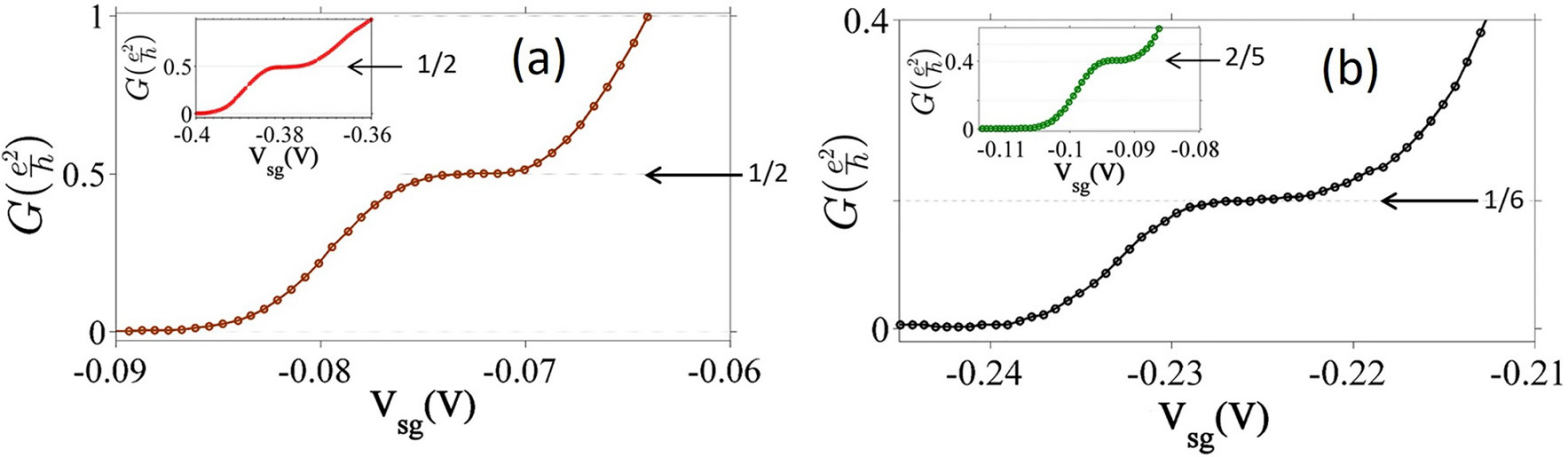
The emergence of fractional conductance plateaus in a tunable confinement potential in a top gated, split gate device. The main plot in (a) and the main and inset plots in (b) show fractional conductance states at 1/2, 1/6, and 2/5, respectively, in an asymmetrically confined 1D quantum wire, and the inset in (a) shows the 1/2 fractional state in a symmetric confinement potential. Adapted from Ref. 25.

**FIG. 10.**
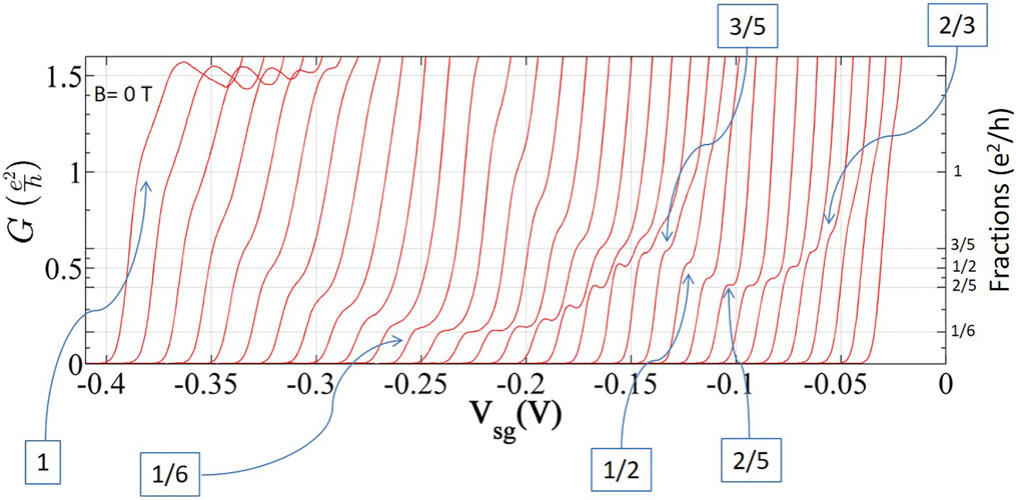
Conductance characteristics of a top-gated split gate device as a function of asymmetry and width of the confinement potential. At V_tg_ = −0.36 V, the offset or asymmetry between the split gates, ΔV_sg_, was incremented from ∼0.65 (left) to 0.97 V (right) in steps of 10 mV, so widening the channel and flattening the confinement. Moving from left, a highly stable fraction at 1/6, was seen with subsequent evolution of fractional states at 1/2, 2/5, 3/5, and 2/3. A horizontal offset of 4 mV was set between the consecutive traces for clarity. Adapted from Ref. 25.

Figure 11 shows the emergence of the 2/5 in (a) and 1/5 in (b) states as a function of decreasing operating temeprature from 2 to 0.02 K. The 2/5 emerges at ∼300 mK, whereas the 1/5 emerges close to 400 mK, only a small change in the carrier concentration is required to switch between the two states. Figure 12(a) is taken from Ref. 95 based on the simulated emergence of the 2/5 as the temperature is reduced from right to left. The horizontal axis in this figure is *μ*/Δ, where *μ* is the electrochemical potential, Fermi energy, and Δ is the fractional gap at the Fermi energy; the similarity between theory and experiment is apparent here. On the formula of Shavit and Oreg,^95^ the number of electrons involved is 3 and 1 for 2/5 and 2 and 1 for 1/5; consequently, the two fractions are closely related and behave in a similar manner as in Fig. 11.

**FIG. 11.**
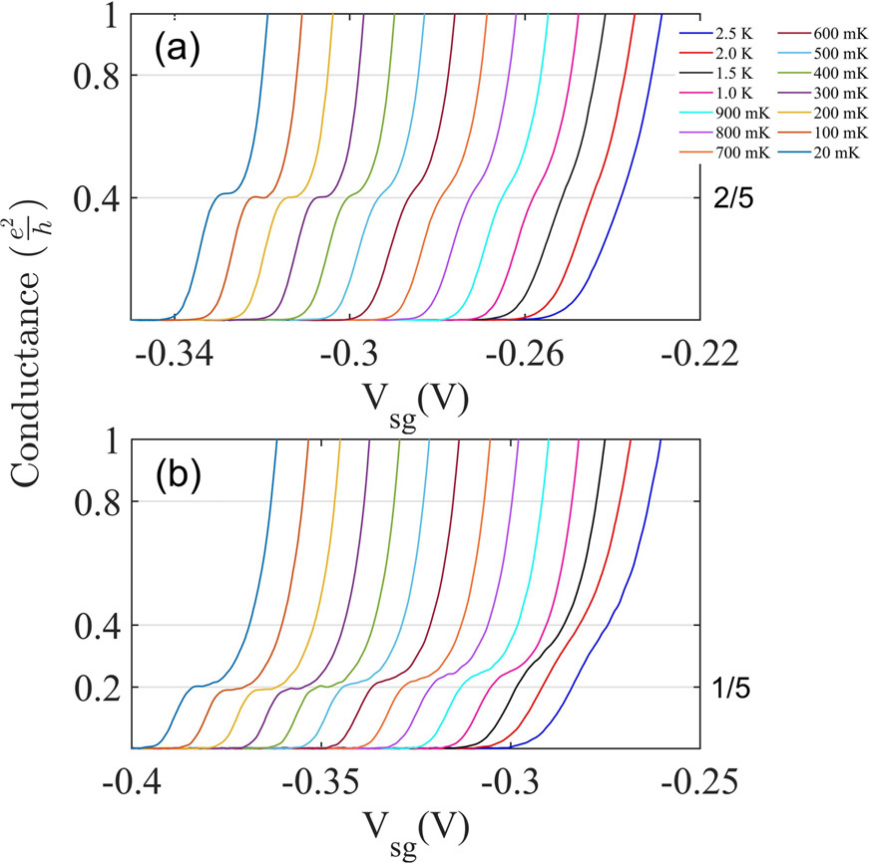
(a) Temperature dependence of the 2/5 fractional state observed in the absence of a magnetic field in a highly asymmetrically defined quantum wire. Adapted with permission from Kumar *et al.*, Appl. Phys. Lett. **115**(12), 123104 (2019). Copyright 2019 American Institute of Physics. (b) Temperature dependence of the 1/5 fractional state observed in a different cooldown to the one shown in Fig. 10. Traces have been offset horizontally in both (a) and (b) for clarity.

**FIG. 12.**
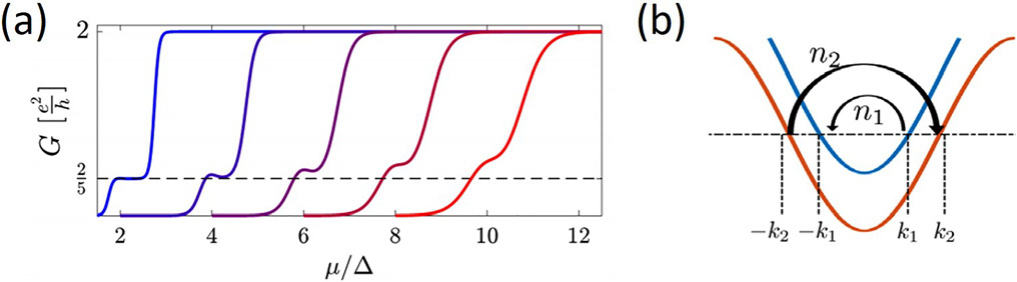
(a) Theoretically simulated conductance for the time reversal invariant nanowire with plateaus at 2/5 ( $\alpha /\sqrt{2m\Delta })$ and at different temperatures (left to right), (T/Δ) = 0.1, 0.2, 0.3, 0.4, and 0.5. Plots are shifted horizontally for clarity. (b) Two-band dispersion with an example of a backscattering process that conserves momentum when the chemical potentials are such that n_1_k_1_ = n_2_k_2_. Adapted with permission from Shavit and Oreg, Phys. Rev. Lett. **123**(3), 036803 (2019). Copyright 2019 American Physical Society.

As previously mentioned, the principal theory of relevance to the experiments shown here is that of Shavit and Oreg,^95^ who have proposed an explanation of the observed non-magnetic odd denominator fractions. They considered the role of momentum conserving coherent backward and forward scattering between two separate lines of carriers, which, in this case, would correspond to the zigzag. The formula they derived for the plateau values is, in units of e^2^/h (*n*_1_ − *n*_2_)^2^/(*n*_1_^2^ + *n*_2_^2^), as illustrated in Fig. 12(b), where *n*_1_ and *n*_2_ are the numbers of particles coherently scattered between bands, so that backscattering in one is counterbalanced by forward scattering in the other and *n*_1_*k*_1_ = *n*_2_*k*_2_, where *k*_1_ and *k*_2_ are the corresponding wave vectors. We immediately see why asymmetric confinement is necessary, so that *n*_1_ and *n*_2_ differ resulting in many fractions; the 2/5 is strong for small numbers of electrons. Weaker plateaus are observed above 1/2, which would correspond to larger numbers of carriers participating a more improbable process. This formula does not predict even fractions, which may have a similar origin as in the FQHE, where they are postulated to result from Bosonic pairing in a BCS model.^96,97^

Insight into the Shavit and Oreg formula can be obtained by considering the scattering process; when the interaction between the two bands is sufficiently strong, they become entangled. If the respective densities of states at the Fermi energy of the two bands are N_1_ and N_2_, then *n*_1_/N_1_ = *n*_2_/N_2_, which follows from the inverse proportionality of momentum and density of states in 1D. The probability of backscattering in the N_1_ band is then N_1_/(N_1_^2^ + N_2_^2^)^1/2^, arising from the entanglement of the two bands, and similarly for the N_2_ band, the probability of forward scattering is N_2_/(N_1_^2^ + N_2_^2^)^1/2^; the probability of the process occurring involving both events is then N_1_N_2_/(N_1_^2^ + N_2_^2^). The scattering directions can be reversed giving a degeneracy of 2, so making use of the relationship *n*_1_/N_1_ = *n*_2_/N_2_, we obtain the probability, P = 2*n*_1_*n*_2_/(*n*_1_^2^ + *n*_2_^2^). The conductance *G* is given by Te^2^/h, where T is the transmission coefficient, normally in ballistic transport T ∼ 1, but in the presence of the backscattering, conductance becomes (1 − P) in units of e^2^/h giving *G* = 1 − 2*n*_1_*n*_2_/(*n*_1_^2^ + *n*_2_^2^). This can be rewritten as (*n*_1_ − *n*_2_)^2^/(*n*_1_^2^ + *n*_2_^2^), which is the result of Shavit and Oreg.^95^

The process of forming the fractions on this model is based on the controlled separation of the carriers as the confinement weakens, and they start to form the zigzag, i.e., a wavefunction with two centers of charge. The carriers do not then behave independently but are coupled or entangled, so resulting in the fractionalization. If the separation of the two rows increases further, then the entanglement breaks down, and the situation may resemble ballistic transport in each row with scattering. When rows are close with strong confinement, they form the normal single wavefunction and a conductance of 2e^2^/h.

It is interesting to note that the fractions found using GaAs could correspond to the fractional charges *fe*, where the conductance is given by (*fe*)*e*/*h*, as in the FQHE. However, the results for the Ge based 1D quantum wire^24^ may be most easily explained on the basis of a conductance given by (*fe*)^2^/*h*, where *f* is given by 1/2 and 1/4, and the 1/2 may correspond to two 1/4 charges that are paired. The difference in the two situations may result from the hole ground state comprising p orbitals resulting in a strongly coupled line.^98^

The differential ac conductance with a dc source-drain voltage that is varied between 0 and ∼−3 mV in the presence of a small signal ac has been used to determine energy levels in a quasi-1D system. The dc voltage drives the system non-Ohmic, and the ac conductance is *G_ac_* = *dI*/*dV_sd_*. When the dc voltage is sufficient to lift the momentum degeneracy, the result is that *G_ac_* takes one half of the Ohmic or linear value. This has been used to investigate energy levels in the integer regime, as the decrease in potential of the 1D subband is given by eV_sd_/2; this can be correlated with the shift in split gate voltage necessary to obtain the plateaus. In the presence of a magnetic field, this technique allows extraction of the Lande g value. The non-Ohmic conductance has been measured for a number of fractions, and the reduction by a factor of 2 has been observed. This suggests that the system is behaving in the same way as in the integer regime. Figure 13 shows the results when a fractional value of 1/2 was found in the Ohmic, linear regime; the data have been moved horizontally for clarity.^25^ The initial rise occurs before the current is unidirectional. The source-drain dc voltage is stepped from 0 on the right to −3 mV on the left. The non-Ohmic plateaus do not quite reach the expected 1/4 possibly due to a change in the carrier concentration, but the main conclusion is that the fractional conductance behaves in the same manner as does the integer.

**FIG. 13.**
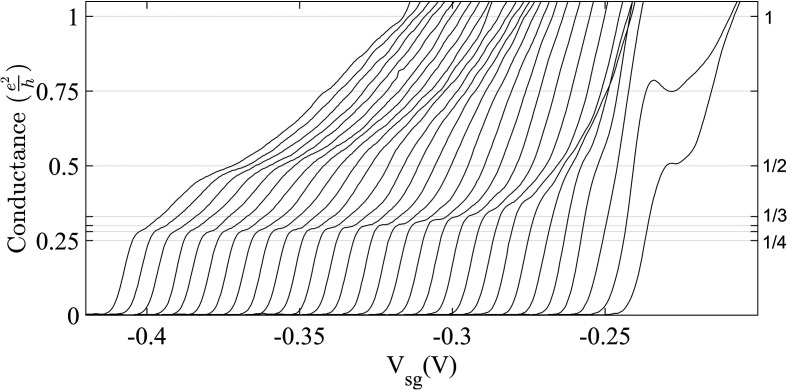
The effect of dc source-drain bias on the differential conductance corresponding to the fractional conductance 1/2 in a symmetrically confined, low density, 1D quantum wire. The source-drain bias, V_sd_, was incremented from 0 (right) to −3 mV (left) in steps of −0.1 mV. Adapted from Ref. 25.

To determine if all the observed fractional plateaus arise from quantum states rather than, for example, a transmission effect, the differential conductance was measured with a measuring signal of 10 *μ*V ac in the presence of 1–3 mV dc. In all cases, the predicted halving of the Ohmic value was obtained. In both Ohmic and non-Ohmic measurements, plateaus of different values but with positive or negative gradient can be found. For example, as shown in Ref. 25 in the presence of a parallel magnetic field, the plateaus shift, and the gradient can increase or decrease, making it important to determine what constitutes a quantum state. A true topological quantum state will maintain its quantized value with changes in the potential environment until the change is sufficiently large to remove the state. We note plateaus in the QHE and FQHE only become flat as the temperature decreases and the magnetic field approaches a particular value; here, the confinement potential produces a similar effect, i.e., the fractional gradient decreases with the approach to the quantized value. Consequently, flatness and accuracy are linked, and a highly accurate value signifies a true quantum state. In addition to the flatness, the data we present here are free of fluctuations and random telegraph signals giving further confidence in the absence of impurity effects.

If current flow is unidirectional, then forward moving electrons will be backscattered into empty states, but there are no backward moving electrons to be scattered into forward moving states. This difficulty implies that there may be a Umklapp processes involved, or the entanglement process is more complex than envisaged to result in cyclical motion of the electrons.

Earlier results presented here show how the ground state changes as the confinement potential is weakened, and a zigzag forms. If this occurs at a low carrier concentration, then the carriers form an entangled entity as they separate, which behaves as if possessing fractional charge. Futher investigations on thermal properties and shot noise to elucidate the effective charge will give a greater understanding of these states.

In conclusion, the field of 1D ballistic transport has matured in the past three decades since its early beginnings with the development of split gate confinement and a variety of new effects have been discovered, in particular the many body aspects.^99^ The ballistic transport in 1D has been well established in III–V compound semiconductors, particularly GaAs, and could be extended into a new class of high quality materials, such as InSb and InAs as well as 2D materials (graphene, bilayer and twisted graphene, silicene, etc.) and topological materials for many body physics. The variety of rich physics the 1D field has to offer can be studied in a range of materials for establishing the robustness of the effects, particularly, the fractional quantized conductance in the absence of magnetic field. One of the striking observations found recently was the self-organization of electrons/holes in the transition between 1D and 2D, resulting in the formation of a zigzag lattice exhibiting fractional conductance plateaus. This unexpected manifestation of interaction effects within the 1D channels indicates that quasi-particles so formed may possess fractional charge—previously only found with the FQHE in 2D systems. The recent experimental results and theory pose further challenges and require new experiments for the estimation of precise electronic charge at the fractional conductance plateaus as well as investigations of entanglement and non-Abelian statistics associated with these fractional states. The physics and technology of 1D quantum structures has much to offer both for fundamental quantum physics of condensed matter and for applications in quantum information schemes.

## Data Availability

The data that support the findings of this study are available from the corresponding author upon reasonable request.
